# Topography of Regional Cerebral GABA_A_ Receptor Availability in Parkinson's Disease Patients With Freezing of Gait

**DOI:** 10.1111/ejn.70618

**Published:** 2026-07-04

**Authors:** Alexander A. Hart, Prabesh Kanel, Jaimie Barr, Giulia Carli, Robert Vangel, Fotini Michalakis, Peter J. H. Scott, Stiven Roytman, Nicolaas I. Bohnen

**Affiliations:** ^1^ Department of Neurology University of Michigan Ann Arbor Michigan USA; ^2^ Department of Radiology University of Michigan Ann Arbor Michigan USA; ^3^ Morris K. Udall Center for Excellence for Parkinson's Disease Research University of Michigan Ann Arbor Michigan USA; ^4^ Parkinson's Foundation Research Center of Excellence University of Michigan Ann Arbor Michigan USA; ^5^ Functional Neuroimaging, Cognitive, and Mobility Laboratory, Department of Radiology University of Michigan Ann Arbor Michigan USA; ^6^ Neurology Service and GRECC VA Ann Arbor Healthcare System Ann Arbor Michigan USA

**Keywords:** cerebellum, freezing of gait, GABA_A_ receptors

## Abstract

We aimed to explore the relationship between regional gamma‐aminobutyric acid (GABA_A_) receptor availability, measured with [^11^C]‐flumazenil brain positron emission tomography (PET) and freezing of gait in patients with Parkinson's disease. Freezing of gait is a significant mobility impairment with limited effectiveness to L‐DOPA in advancing disease implying a role for other neurotransmitters, such as GABA. Imaging studies using [^11^C]‐flumazenil PET and magnetic resonance imaging (MRI) were conducted in 33 patients with Parkinson's disease (9F/24M; age 68.34 ± 6.38, disease duration 8.03 ± 4.73, motor Movement Disorders Society‐revised Unified Parkinson's Disease Rating Scale (MDS‐UPDRS) scores 44.01 ± 14.85). Patients were classified into two groups: “freezers” (*n* = 8) and “nonfreezers” (*n* = 25), based on the MDS‐UPDRS Part III off state examination. Whole brain voxel‐based *t*‐tests group comparisons were performed using SPM12. Reduced GABA_A_ binding was observed in the cerebellum vermis, esp. vermis lobule VI, left posterior cingulum, posterior parahippocampal gyrus/fimbriae, medial occipital–temporal gyrus and the right gyrus rectus, right anterior cingulum, and adjacent right superior frontal gyrus that demonstrated significantly reduced GABA_A_ receptor availability in the individuals with freezing as compared to those without. In addition, reductions were also seen in the left posterior putamen and pallidum. Findings may augur a role for GABA_A_ inverse agonists for novel investigation of FOG treatment in Parkinson's disease.

## Graphical abstract

Reduced gamma‐aminobutyric acid (GABA_A_) receptor availability may augur a role for GABA_A_ inverse agonists for treatment of freezing of gait in Parkinson's disease.

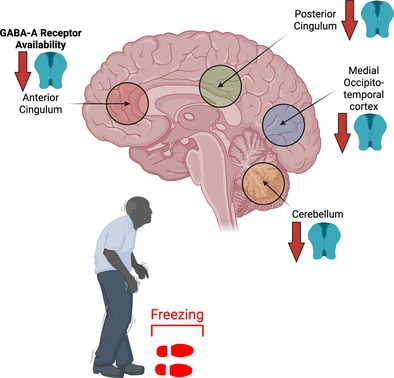

**Abbreviations**
AUC: Area under the curve
DTBZ: [^11^C]‐dihydrotetrabenazine
DTI: Diffusion tensor imaging
FEOBV: [F^18^]fluoroethoxybenzovesamicol
fMRI: functional magnetic resonance imaging
FMZ: [^11^C]‐flumazenil
FoG: freezing of gait
FWHM: Full width at half maximum
GABA: Gamma‐aminobutyric acid
HY: Hoehn and Yahr scale
MDS‐UPDRS: Movement Disorders Society‐revised Unified Parkinson's Disease Rating Scale
MNI: Montreal Neurological Institute
MRI: Magnetic resonance imaging
MRS: Magnetic resonance spectroscopy
PD: Parkinson's disease
PET: Positron emission tomography
PIGD: Postural instability and gait disorders
ROC: Receiver operating characteristic
SPM: Statistical Parametric Mapping
VAChT: Vesicular acetylcholine transporter

## 1 Introduction

Postural instability and gait disorders (PIGD) can lead to significant mobility impairments in persons with Parkinson's disease (PD), such as freezing of gait (FoG) in 44% and falls in 70% of patients (Contreras and Grandas 2012b, 2012a). While FoG and falls may be interconnected episodic phenomena in PD, recent studies have shown that there are partial neurobiological differences in their pathophysiology (Bloem et al. 2004). One such difference noted in a brain vesicular acetylcholine transporter (VAChT) [F^18^]fluoroethoxybenzovesamicol (FEOBV) positron emission tomography (PET) study found that cholinergic deficits in the thalamus were associated with falls while striatal, limbic (amygdala, hippocampal), and cortical deficits were associated with FoG (Bohnen et al. 2019). Furthermore, the cerebellum has also been implicated in FoG physiology and overall disordered gait in PD in multiple neuroimaging studies (Jung et al. 2020; Wu and Hallett 2013).

Gamma‐aminobutyric acid (GABA) is an inhibitory neurotransmitter found in sensorimotor circuits important to gait in general as well as in PD (Boecker et al. 2010; O'Gorman Tuura et al. 2018; Petroff 2002). GABA_A_ specifically is a pentameric ligand‐gated ion channel that binds with the neurotransmitter GABA leading to postsynaptic inhibition and is a common target in other realms of medicine, such as anxiety, sleep disorders, and epilepsy (Zhu et al. 2022). While the contribution of GABA to specific motor and gait deficits in PD is understudied, there has been some work which has found that GABA levels are lower in the left basal ganglia as compared to controls in humans with further studies identifying a role of GABAergic transmission in PD mouse models (Gong et al. 2018; Liu et al. 2019; Mograbi et al. 2017). Given this prior role, investigators have explored flumazenil pharmacotherapy in persons with PD and found post administration improvements in tapping speed and Movement Disorders Society‐revised Unified Parkinson's Disease Rating Scale (MDS‐UPDRS) scores that further support the role of GABA in PD physiology (Ondo and Hunter 2003).

As described above, the precise role of the GABAergic system in mobility, and particularly FoG in PD, remains unclear. To further elucidate this relationship, this study aimed to explore the regional relationship between GABA_A_ receptor availability with [^11^C]‐flumazenil (FMZ) PET and FoG in persons with PD to identify areas of altered GABAergic transmission. We hypothesized that topographic GABA_A_ receptor availability changes across large scale neural circuitry may associate with FoG in PD.

## 2 Materials and Methods

### 2.1 Subjects

This cross‐sectional study included 33 subjects diagnosed with PD according to the UK Parkinson's Disease Society Brain Bank clinical criteria (Hughes et al. 1992). The presence of nigrostriatal dopaminergic denervation was confirmed using vesicular monoamine transporter type 2 [^11^C]‐dihydrotetrabenazine (DTBZ) PET imaging. Additional inclusion criteria include a Hoehn and Yahr scale (HY) of 2 to 4. The exclusion criteria included a history of large artery stroke or other significant intracranial pathology. Motor severity was assessed by an examiner using the modified HY and the MDS‐UPDRS Part III. Clinical examinations and PET imaging were performed in the morning following an overnight withdrawal of dopaminergic medications in “off” state. At the time of the study, no participants were taking benzodiazepines, GABA_B_ ‐ergic medication (baclofen and tizanidine), modafinil, neuroleptics, anticholinergics (trihexyphenidyl and benztropine), or cholinesterase inhibitor drugs. Patients were classified into two groups: “freezers” and “nonfreezers”, based on the subsection of MDS‐UPDRS Part III motor examination (3.11). The study protocol was approved by the Institutional Review Boards of the University of Michigan and the Ann Arbor VA Medical Center in accordance with the Declaration of Helsinki, and all subjects provided written informed consent.

### 2.2 Imaging Techniques

Participants underwent a brain MRI using three Tesla Philips Achieva system (Philips, Best, The Netherlands) and two different PET scans using Biograph 6 TruPoint PET/CT scanner (Siemens Molecular Imaging Inc., Knoxville, TN) as previously described (Kanel et al. 2022). PET [^11^C]‐flumazenil ([^11^C]‐FMZ) PET imaging was used to examine GABA_A_ receptor availability [^11^C]‐FMZ. PET was synthesized according to established protocols (Frey et al. 1991; Koeppe et al. 1999). [^11^C]‐FMZ (10 mCi) was re‐administered using a bolus‐plus‐infusion method. In FMZ, dynamic PET imaging was performed with an intravenous bolus injection containing 40% of the total administered dose over 15 s, followed by a continuous infusion of the remaining tracer at a constant rate for 62 min (Frey et al. 1991). Similarly, in DTBZ, dynamic PET imaging was performed with an intravenous bolus injection containing 55% of the total administered dosage, with the remaining dosage delivered by continuous infusion of the remaining tracers over the next 60 min (Koeppe et al. 1999). All imaging procedures were completed within a few days, with two PET scans typically conducted on the same day.

### 2.3 Imaging Analysis and Spatial Preprocessing

All images underwent spatial coregistration via rigid‐body transformation to correct for subject movement. To create a parametric image, we computed the distribution volume ratio using Logan graphical analysis (Logan et al. 1996) using the anterior pontine reference region serving as the reference region due to negligible specific binding (Millet et al. 2002; Odano et al. 2009).

We used SPM12 to segment structural MRIs and applied the Muller–Gartner method to correct for partial volume effects in both the parametric PET images (Müller‐Gärtner et al. 1992). Both the MRI and corrected parametric PET scans were then normalized to a study‐specific MNI template using DARTEL. To enhance the signal‐to‐noise ratio, we applied an 8‐mm full width at half maximum (FWHM) spatial smoothing kernel to the final PET data in MNI space.

### 2.4 Statistical Analysis

Voxel‐based statistical analysis was performed using Statistical parametric mapping (SPM12) software (Wellcome Trust Centre for Neuroimaging, University College, London, England [https://www.fil.ion.ucl.ac.uk/spm/software/spm12/]). Two‐sample *t*‐test was performed on FMZ PET data in Montreal Neurological Institute (MNI) space, comparing two groups: one group consisting of individuals with PD with FOG and the other group comprising individuals with PD without FoG. Sex and disease duration since the onset of the first symptom were included as nuisance covariates in the analysis. We examined both positive and negative t‐maps, applying a voxel‐level threshold of *p* < 0.001. Significance was determined using cluster‐level family‐wise error (FWE) correction, with a threshold of *p* < 0.05 to account for multiple comparisons across the entire brain.

To evaluate whether the observed association was robust, the mean DVR for each subject was extracted only from the statistically significant clusters found in our SPM results. The open‐source *R caret* package was used to perform leave‐one‐out cross‐validated logistic regression to predict freezing status from cluster DVR. Hold‐out predictions from each fold were aggregated to compute a cross‐validated receiver operating characteristic (ROC) curve, along with area under the curve (AUC), sensitivity, and specificity, using the *pROC* package.

## 3 Results

### 3.1 Clinical Findings

A total of 33 participants were included in this study. Of those, 24 (72.7%) were male and 9 (27.2%) were female, with an overall mean age of 68.3 years old (standard deviation of 14.9 years). In terms of PD, the mean disease duration was 8.7 years (standard deviation of 4.7 years) with an average MDS‐UPDRS score of 44.0 (standard deviation of 14.9), modified HY (Stage 2: 5; Stage 2.5: 12; Stage 3: 14; Stage 4: 2). In terms of FoG, 8 (24.2%) were in the “freezers” category and 25 (75.8%) in the nonfreezer category.

### 3.2 Imaging Findings

**FIGURE 1:**
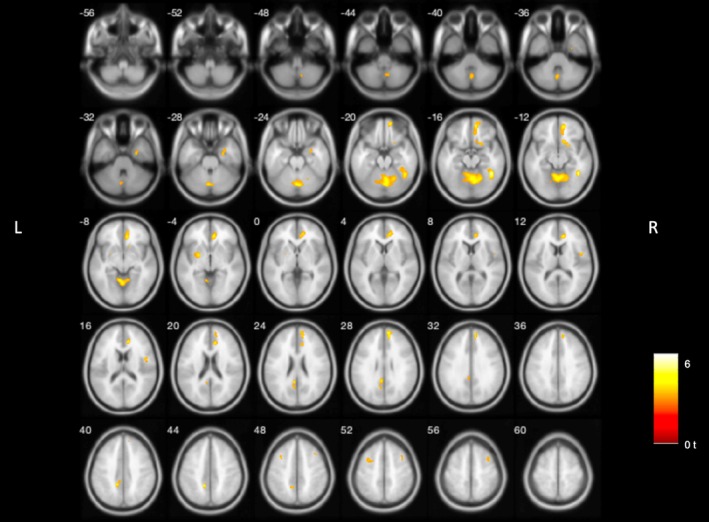
Differences in FMZ binding between PD patients with FoG and those without FoG in MNI space. Compared to PD without FoG, voxel‐based analyses revealed lower FMZ binding in the cerebellum and vermis. Additional reductions were noted in both cortical and subcortical regions, specifically in the fusiform gyrus, lingual gyrus, left parahippocampal gyrus, right anterior cingulate cortex, left posterior cingulate cortex, right superior frontal gyrus, right orbital frontal cortex, and right gyrus rectus. (*p* < 0.001; clusterwise FWE‐corrected).

Our SPM12‐based voxel‐based analysis showed that individuals with PD and FOG exhibited significantly reduced GABA_A_ binding compared to those without FOG (FWE‐corrected at *p* < 0.05; see Figure 1; Table 1). The cerebellum vermis, esp. vermis lobule VI, left posterior cingulum, the posterior parahippocampal gyrus/fimbriae, medial occipital‐temporal gyrus and the right gyrus rectus, right anterior cingulum, and adjacent right superior frontal gyrus demonstrated significantly reduced GABA_A_ density in the individuals with FoG as compared to those without. In addition, reductions were also seen in the left posterior putamen and pallidum. A detailed cluster analysis with significant clusters is present in Table 1.

**TABLE 1:** Clusters of significantly reduced [^11^C]‐GABA_A_ binding in the PD subjects with freezing of gait compared to PD subjects without freezing of gait.

	Peak MNI coordinates						
Cluster (voxels)	*X*	*Y*	*Z*	BA	Peak *t*‐value	Peak voxel location	Predominant network hub
1572	6	−66	−18	19	5.34	Lobule VI of vermis	Bilateral lobules IV, V, VI, and VIII of cerebellar hemisphere Bilateral lingual gyrus Left Crus I and Crus II of cerebellar hemisphere Right fusiform gyrus Right lobule IX of cerebellar hemisphere Lobules IV, V, VI, VII, VIII, and IX of vermis
84	28	−8	−30		4.03	Right parahippocampal gyrus	Right parahippocampal gyrus Right hippocampus Right amygdala
261	42	−50	−14	20,37	6.84	Right inferior temporal gyrus	Right inferior temporal gyrus Right fusiform
571	12	50	−14	10,11,24,32	4.98	Right superior frontal gyrus	Right medial frontal gyrus Right anterior cingulate cortex Right rectus Right orbital frontal Gyrus Right superior frontal gyrus
96	12	18	−12	47	4.24	Right rectus	Right rectus Right olfactory
83	−24	−2	−4		4.49	Left pallidum	Left putamen Left pallidum
59	48	2	14		4.34	Right rolandic operculum	Right rolandic operculum
159	10	54	28	9	4.84	Right superior frontal gyrus	Right superior frontal gyrus Right anterior cingulate cortex
97	−6	−46	26	31	4.77	Left post cingulate	Left precuneus Left post cingulate
74	−14	−44	42	7,31	5.44	Left precuneus	Left precuneus Left middle cingulum
82	−34	6	52	6	4.07	Left middle frontal gyrus	Left middle frontal gyrus
50	36	8	56	6	3.77	Right middle frontal gyrus	Right middle frontal gyrus

*Note:* Results are based on SPM voxel‐based analysis, adjusted for sex, [^11^C] DTBZ binding in the striatum and disease duration from the first symptom, and corrected for multiple comparisons using cluster‐level FWE correction (*p* < 0.001, FWE of < 0.05; minimum cluster size: 50 voxels; Table 1).

Leave‐one‐out cross‐validated logistic regressions yielded a ROC curve with an AUC of 0.85, sensitivity of 0.96, and specificity of 0.62, thus suggesting that GABA_A_ receptor binding is a robustly sensitive predictor of freezing status.

## 4 Discussion

Our study examined 33 participants with PD with a mean UPDRS score of 44 who underwent ^11^[C]‐flumazenil PET scan comparing 8 individuals with FoG to 25 without FoG. We identified the cerebellum, esp. vermis lobules IV and V, the posterior parahippocampal gyrus/fimbriae, medial occipital‐temporal gyrus, and the right gyrus rectus, right anterior cingulum, left posterior cingulum, and adjacent right superior frontal gyrus to have significantly reduced GABA_A_ receptor availability in the individuals with FoG as compared to those without. To date, there are no specific findings linking GABA magnetic resonance spectroscopy (MRS) studies directly with PD patients with FoG. The available MRS literature on GABA generally focuses on axial symptoms but without specific assessment of FoG (Piras et al. 2020). PD patients exhibit significantly higher GABA levels in the basal ganglia compared to healthy controls. The elevated basal ganglia GABA levels correlate positively with the overall degree of gait disturbance in PD patients, especially in akinetic‐rigid compared to tremor‐predominant patients. A cerebellar GABA MRS study reported association with cognitive interference but not on FoG (Piras et al. 2020).

The cerebellum, and in particular the cerebellar vermis, has been implicated in gait in general and gait dysfunction in PD with the suspicion that FoG could be related to impairment of the functional network connectivity to the cerebellar locomotor region (Fasano et al. 2017). This relationship has been identified, using functional MRI (fMRI) technology between 38 persons with PD with FoG compared to 17 without freezing and showing increased connectivity between the cerebellar vermis and multiple cortical areas including the superior parietal lobes, supplemental motor area, and precentral gyrus, while there was reduced connectivity to the occipital lobe, which was hypothesized to an adaptive response to reduced automaticity of gait and increased reliance of visual input for gait (Lench et al. 2025). Additional work has shown overall reduced functional activation of the cerebellar vermis while completing motor and visual imagery tasks in PD freezers compared to nonfreezers (Dijkstra et al. 2023). Utilizing fMRI imaging and diffusion tensor imaging (DTI) examining cerebellar peduncles dysfunction in this area was found to be proportional to severity of FoG (Bharti et al. 2019). Overall, it has been demonstrated that in the setting of FoG cerebellar connectivity from the cerebellar, vermis has been implicated consistent with the results of our study. The cerebellar vermis is also strongly connected with the various lobules of the cerebellum, and these connections are vital in the maintenance of balance and perceptual stability (Cullen 2023). The vermis also connects to the lingual gyrus where reduced cerebral blood flow has been be associated with cognitive and gait impairment in individuals with normal pressure hydrocephalus (Suzuki et al. 2022). Expanding on this functional connectivity, studies in PD have demonstrated evidence of impaired connectivity to the lingual gyrus in those with gait difficulty and implying that this could relate to impairment of visuomotor integration (Pellegrini et al. 2024). Our results further support prior work, such as that by Li et al. who found in a cohort of patients with PD alterations in spontaneous activity in both the cerebellar vermis and fusiform gyrus in individuals with FoG (Licen et al. 2022).

Similar to the cerebellum, the gyrus rectus has been previously implicated to gait abnormalities in PD (Bohnen et al. 2024). This structure is highly integrated with the entorhinal cortex and hippocampus and is postulated to be involved in higher level processing as a possible mechanistic explanation for how it could contribute to gait dysfunction (Karaca et al. 2025). It notably has been found to be altered among individuals with FoG, such as in the study by Li et al. who found abnormal topological changes to the gyrus rectus in 20 individuals with PD and FoG who were followed for 5 years implicating disrupted regional topological organization contribution (Li et al. 2022). Similarly, a MRI study completed by Wang et al. identified reduced network efficiency in the gyrus rectus, supplementary motor areas, and middle cingulate cortex in those with FoG with both structural and network abnormalities identified (L. Wang et al. 2023). Furthermore, on cholinergic imaging, this area was found to have reduced cholinergic binding identified abnormalities in the gyrus rectus to be associated with balance abnormalities in PD (Bohnen et al. 2024).

The anterior and posterior cingulate cortices were also identified to have reduced GABA_A_ receptor binding. Prior work by Bohnen et al. utilizing VAChT brain PET identified the cholinergic centro‐cingulate network to be altered in PD (Bohnen et al. 2023). They identified that cholinergic losses in this system, with the cingulum being a core neuroanatomical component, result in significant changes in overall cognition as well as gait, including FoG and falls in PD. Through other modalities, this has also been supported on structural MRI studies where reduced gray matter thickness in both the anterior and posterior cingulate has been associated with FoG (Tessitore et al. 2012; Vastik et al. 2017). Similarly in fMRI studies, the right anterior cingulate cortex reduced spontaneous activity that was associated with FoG in PD. Returning to prior concepts, the cingulate cortex and limbic systems have been associated via network connections with the ventromedial prefrontal cortex, implicating the gyrus rectus as a possible connection as well (Rolls 2019).

In anatomically related areas, the parahippocampal gyrus and medial and posterior inferior temporal lobes were also identified as structures with abnormal GABA_A_ receptor binding in our study. These structures are heavily interconnected with the limbic circuits and could mechanistically relate to gait through impairment of spatial memory (Rajmohan and Mohandas 2007; Squire et al. 2004). There has been further work completed by LaFlamme et al. in macaque monkey models where they identified that the functional connectivity between the hippocampus and the parahippocampal cortex is vital for spatial memory and ultimately gait (LaFlamme et al. 2021; X. Wang et al. 2017). In the setting of FoG, these areas have also been implicated prior. For example, Gan et al. using structural MRI found that FoG was significantly associated with reduced local gyrification index of the left entorhinal cortex and parahippocampal gyrus (Gan et al. 2023). Additionally, using fMRI in another study, it was also found that there was decreased connectivity in the parahippocampal gyrus and inferior temporal gyrus among persons with PD with FoG (Jin et al. 2021).

In addition to the above, there were several other locations associated with FoG. The globus pallidus pars internus and putamen were associated, which notably have been used previously as deep brain stimulation targets as a therapeutic approach for FoG, supporting its role in this entity (Lee et al. 2022; Schrader et al. 2011; Wong et al. 2021). Additional areas were also identified to have reduced GABA_A_ receptor binding, such as the middle frontal regions, superior frontal gyrus, and temporal operculum. Interestingly, no brainstem or thalamic regions were identified in our study.

This study is not without limitations. The largest being a relatively small sample size with a low percentage of freezers (24.2%) limiting statistical power. Future studies using larger sample sizes are needed to confirm our observation. Importantly, this relatively small sample size should be considered when applying these results as it may affect the generalizability of this work. Other primary limitation is that assessment of FoG was only based on the examiner assessment of FoG during the MDS‐UPDRS motor examination and not using a dedicated FoG provocation test battery assessment. This limits the sensitivity of this study to identify FoG. However, the significant group differences indicate that our assessment is conservative as the labeled non‐FoG group may include patients with less severe FoG that might have been recognized in a dedicated FoG provocation protocol. Thus, the MDS‐UPDRS is a highly specific but less sensitive diagnostic assessment and may miss mild FoG in the control group diluting our findings. Therefore, future studies should be based on specialized FoG provocation test protocols and aided by sensor‐based FoG detecting technologies.

## 5 Conclusions

This study examined a cohort of persons with PD with FMZ PET to assess areas of altered GABA_A_ receptor availability in individuals with FoG versus those without. It was found that the cerebellum, esp. vermis lobule VI, the posterior parahippocampal gyrus/fimbriae, medial occipital‐temporal gyrus and the right gyrus rectus, right anterior cingulum, and adjacent right superior frontal gyrus had reduced GABA_A_ receptor availability in those with FoG. While this study is limited by sample size, it demonstrates evidence of GABAergic changes in the brains of PD freezers that is compatible with prior FEOBV PET, structural MRI, and fMRI studies. Further work should be done with larger cohort sizes to validate these results. Within the described limitations, these findings could augur a role for GABA_A_ reverse agonists in the management of FoG in PD.

## Author Contributions

**Alexander A. Hart:** conceptualization, writing – original draft, writing – review and editing. **Prabesh Kanel:** conceptualization, formal analysis, data curation, methodology, software, visualization, writing – review and editing. **Jaimie Barr:** writing – review and editing. **Giulia Carli:** writing – review and editing. **Robert Vangel:** writing – review and editing. **Fotini Michalakis:** writing – review and editing. **Peter J. H. Scott:** writing – review and editing. **Stiven Roytman:** writing – review and editing. **Nicolaas I. Bohnen:** conceptualization, methodology, funding acquisition, supervision, investigation, project administration, writing – review and editing.

## Funding

This research was supported by the National Institutes of Health (grant numbers P01 NS015655 and NS099535) & the U.S. Department of Veterans Affairs (grant number I01 RX000317).

## Conflicts of Interest

The authors declare no conflicts of interest.

## Data Availability

The dataset used and analyzed during the present study can be made available by the corresponding author or last author upon reasonable request from qualified researchers. Because the dataset includes highly personal data, access will require a formal data‐sharing agreement approved by the relevant institutional ethics bodies.
